# Personal radio use and cancer risks among 48,518 British police officers and staff from the Airwave Health Monitoring Study

**DOI:** 10.1038/s41416-018-0365-6

**Published:** 2018-12-26

**Authors:** He Gao, Maria Aresu, Anne-Claire Vergnaud, Dennis McRobie, Jeanette Spear, Andy Heard, Håvard Wahl Kongsgård, Deepa Singh, David C. Muller, Paul Elliott

**Affiliations:** 1 Department of Epidemiology and Biostatistics, School of Public Health, Imperial College London, London, UK; 2 Faculty of Medicine, Norwegian University of Science and Technology (NTNU), Trondheim, Norway; 3 MRC-PHE Centre for Environment and Health, Department of Epidemiology and Biostatistics, School of Public Health, Imperial College London, London, UK; 4 NIHR Health Protection Research Unit in Health Effects of Environmental Hazards, Imperial College London, London, UK; 5 UK Dementia Research Institute (DRI) at Imperial College, Imperial College London, London, UK; 6 Health Data Research-UK (HDR) London Centre at Imperial College, Imperial College London, London, UK

**Keywords:** Epidemiology, Cancer

## Abstract

**Background:**

Radiofrequency electromagnetic fields (RF-EMF) from mobile phones have been classified as potentially carcinogenic. No study has investigated use of Terrestrial Trunked Radio (TETRA), a source of RF-EMF with wide occupational use, and cancer risks.

**Methods:**

We investigated association of monthly personal radio use and risk of cancer using Cox proportional hazards regression among 48,518 police officers and staff of the Airwave Health Monitoring Study in Great Britain.

**Results:**

During median follow-up of 5.9 years, 716 incident cancer cases were identified. Among users, the median of the average monthly duration of use in the year prior to enrolment was 30.5  min (inter-quartile range 8.1, 68.1). Overall, there was no association between personal radio use and risk of all cancers (hazard ratio [HR] = 0.98, 95% confidence interval [CI]: 0.93, 1.03). For head and neck cancers HR = 0.72 (95% CI: 0.30, 1.70) among personal radio users vs non-users, and among users it was 1.06 (95% CI: 0.91, 1.23) per doubling of minutes of personal radio use.

**Conclusions:**

With the limited follow-up to date, we found no evidence of association of personal radio use with cancer risk. Continued follow-up of the cohort is warranted.

## Introduction

Since the widespread dissemination of mobile telephones and other wireless devices in the 1990s, there has been both public concern and scientific debate regarding exposure to radiofrequency (RF) electromagnetic fields (EMF) emitted from these devices and possible effects on health, especially cancer. The International Agency for Research on Cancer (IARC) on reviewing the totality of evidence classified RF-EMF as possibly carcinogenic to humans (Group 2B).^1^ Terrestrial Trunked Radio (TETRA) is used for radio communications among British police forces and other emergency services. The average output power of TETRA radios can, in some circumstances, exceed that from mobile phones and as compared with mobile phones, for which transmission is pulsed at 217 Hz, the TETRA signal has a pulse rate of 17.6  Hz, so the mechanism of any potential effects on health including cancers may differ. In addition, no RF-EMF is emitted by the radio in listening mode. In 2000, the UK Independent Expert Group on Mobile Phones (Stewart Report) suggested that as a precautionary measure signal modulation around 16 Hz should be avoided if possible in future signal coding development^2^ based on experimental findings of increased brain calcium efflux associated with such frequencies in animal models.^3^ To address this concern, the Home Office commissioned the Airwave Health Monitoring Study to assess possible long-term health effects of TETRA use among the British police. We present here the first report from the study of cancer risks associated with TETRA use.

## Methods

### Study population

The Airwave Health Monitoring Study is an occupational cohort launched in June 2004 enrolling police personnel across Great Britain. The study design and rationale have been described previously.^4^ A total of 53,114 participants were enrolled by end of recruitment in March 2015. Personal radio usage information was available for 49,286 participants. We excluded a further 768 participants with a prior cancer diagnosis from cancer registry data or on questionnaire, leaving 48,518 participants (91% of total cohort sample) for this analysis (Supplementary Figure 1).

### Radio use

Personal radio (issued to individual officers and staff) was the TETRA device most commonly used in the study. For our main exposure variable, we estimated average monthly personal radio call duration (in minutes) during the year prior to enrolment. Objective data on TETRA use from computerized records linked to personal radio users (call data record [CDR] database) were used if available, as previously described.^5,6^ When objective records were not found, we assigned personal radio usage at enrolment to zero for self-reported non-users and, for radio users, we applied multiple missing value imputation to estimate duration of use in the year before enrolment, based on self-reported data and participant characteristics at enrolment.^6^ The imputation substantially reduced exposure mis-classification compared with self-reported data; details are available elsewhere.^5,6^

In addition, in secondary analyses, for participants who could be linked to their CDR records, we computed average monthly personal radio duration of use for each year from all the available yearly periods before enrolment (range up to 10 years, median 3 years, inter-quartile range [IQR] 2–5 years), and then took the average of those data to calculate average duration of use in minutes/month. Where there was a gap in use of more than one year (*N* = 241, 1.2% of CDR linked personal radio users), we only considered the most recent consecutive yearly periods before enrolment to calculate average duration of use. This is because it was possible that there were different users of the radio before and after the gap in data.

### Cancer outcomes

We used record linkage to obtain cancer events and deaths from national registries. Cancer diagnoses were coded using International Classification of Diseases (ICD)-9/ICD-10 versions for events before/after 1998 up to 30 June 2016 for England and Wales and 31 Dec 2013 for Scotland. Person-time at risk, started at study enrolment, was accrued until date of first cancer diagnosis, death, or end of administrative follow-up, whichever occurred first. We defined “all cancers” as malignant neoplasm (MN) across all sites except non-melanoma skin cancer, for which data are incomplete or inconsistent in the registries. Because the police wear the personal radio device on the upper chest with the aerial in proximity to the head and neck when speaking, we separately considered “head and neck cancers” based on the National Cancer Intelligence Network definition (http://www.ncin.org.uk/view?rid=2108) with addition of MN of eye and adnexa (C69), MN of brain (C71), MN of spinal cord, and cranial nerves (C72) (Supplementary Table 2).

### Statistical analysis`

We used Cox proportional hazards model to compute hazard ratios (HRs) for all cancer and head and neck cancers in relation to estimated monthly personal radio usage in the year before enrolment. Age was used as the underlying time scale with adjustment for potential confounders of sex, region, education, salary, rank, job satisfaction, body mass index (BMI), smoking, number of cigarettes smoked, and alcohol drinking. Among personal radio users, we log_2_ transformed their usage in minutes per month, so HRs correspond to a doubling in usage time. We included an interaction term with a binary variable for personal radio user (yes/no), which gave us an estimate for the hazard of being a radio user compared with non-user. The proportional hazards assumption was investigated via graphical inspection of smoothed, scaled Schoenfeld residuals. We repeated the analyses restricting the sample to police officers, who represent the majority of personal radio users and have a higher usage level than staff. Sex-stratified analyses were also performed except for head and neck cancers among female officers due to the small number of cancer cases in that group. We also carried out a sensitivity analysis excluding forces with less than 5% of objective data among personal radio users (Supplementary Figure 1). In this restricted sample, we further calculated HRs for all cancers using average monthly personal radio use derived from all the available data before enrolment for those participants with CDR data (Supplementary Figure 1).

## Results

Participant characteristics are shown in Supplementary Table 1: 17,725 (36.5%) participants were female, 32,369 (66.7%) were a personal radio user, and 31,255 (64.4%) were officers. Average age at enrolment was 40.3 years (SD 9.1); median personal radio usage among users was 30.5 min per month (IQR: 8.1, 68.1). Median follow-up time from enrolment was 5.9 years (IQR: 3.4, 8.6). There were 716 incident cancer cases during an accumulated follow-up of 290,617 person-years, including 74 head and neck cancers (22 brain tumours; Supplementary Table 2).

Overall, there was no association between personal radio use and risk of all cancers (HR = 1.10, 95% confidence interval [CI]: 0.84, 1.44, users compared with non-users, Table 1). In analyses restricted to police officers, the estimated HR was 0.81 (95% CI: 0.56, 1.17); the reduced risk was largely driven by results for male officers, though estimated HRs had considerable uncertainty. Doubling of monthly personal radio call duration among users was not associated with all-cancer risk either for all users or when only officers were considered (Table 1).

**Table 1:** Hazard ratios of personal radio use for all cancers and head and neck cancers

	All (*N* = 48,518)			Males (*N* = 30,793)			Females (*N* = 17,725)		
	*N* cases	HR (95% CI)	*P*-value	*N* cases	HR (95% CI)	*P*-value	*N* cases	HR (95% CI)	*P*-value
All cancers^a^									
Non-user	343	1.00		154	1.00		189	1.00	
User	373	1.10 (0.84–1.44)	0.492	252	0.93 (0.66–1.31)	0.683	121	1.30 (0.84–2.02)	0.247
Doubling of minutes of use		0.98 (0.93–1.03)	0.337		0.98 (0.92–1.04)	0.496		1.00 (0.91–1.09)	0.940
Head and neck cancers^b^									
Non-user	26	1.00		12	1.00		14	1.00	
User	48	0.72 (0.30–1.70)	0.449	38	0.72 (0.26–1.98)	0.524	10	0.62 (0.10–3.92)	0.608
Doubling of minutes of use		1.06 (0.91–1.23)	0.444		1.04 (0.88–1.23)	0.636		1.17 (0.82–1.67)	0.390
	**All officers (** ***N*** ** = 31,255)**			**Male officers (** ***N*** ** = 23,240)**			**Female officers (** ***N*** ** = 8015)**		
All cancers^a^									
Non–user	79	1.00		54	1.00		25	1.00	
User	239	0.81 (0.56–1.17)	0.262	171	0.76 (0.49–1.19)	0.235	68	0.99 (0.51–1.92)	0.983
Doubling of minutes of use		1.01 (0.94–1.07)	0.869		1.01 (0.93–1.09)	0.897		1.01 (0.90–1.13)	0.874
Head and neck cancers^b^									
Non-user	9	1.00		9	1.00		–	–	–
User	36	0.71 (0.25–2.04)	0.527	31	0.60 (0.20–1.81)	0.363	–	–	–
Doubling of minutes of use		1.05 (0.89–1.25)	0.538		1.04 (0.87–1.25)	0.640	–	–	–

*HR* hazard ratio

^a^All cancers include all the sites except for non-malignant melanoma (ICD10 = C44)

^b^Head and neck cancers include malignant neoplasm (MN) of lip, oral cavity, and pharynx (C00–C14), MN of nasal cavity and middle ear (C30), MN of larynx (C32), MN of eye and adnexa (C69), MN of brain (C71), MN of spinal cord, cranial nerves (C72), and MN of thyroid gland (C73)

The model adjusted for age (the underlying time scale), sex, region, education, salary, rank, job satisfaction, BMI, smoking, number of cigarettes smoked, and alcohol drinking. The HR for usual personal radio use represents the increase in risk for a doubling of personal radio use (average number of minutes per month)

The estimated hazard of developing head and neck cancers was 0.72 (95% CI: 0.30, 1.70), i.e., 28% lower among personal radio users as compared with non-users, and among users it was 1.06 (95% CI: 0.91, 1.23) per doubling in minutes of personal radio use, although both estimates were again accompanied by substantial uncertainty. Similar estimates were obtained when restricting the analysis to police officers (Table 1).

In the sensitivity analysis for forces where at least 5% of the personal radio users could be linked to the CDR database (*N* = 38,888), the results were largely consistent with the main analysis. There was some weak evidence of an association between radio use and lower risk of all cancers, but only for the all-officer analysis (HR = 0.67 [95% CI: 0.45, 1.00]; Supplementary Table 3). Comparing users and non-users among officers only, the non-users were on average older (44.4 vs 38.7 years, respectively), were more obese, were more likely to be heavier drinkers of alcohol, and had lower levels of education (Supplementary Table 4); 20% of the non-users were previous users of the radio. When personal radio users were further restricted to those who could be linked to the CDR database over one or more consecutive years before enrolment, there were 32,330 participants and 259 incident cases of all cancers. No association with all-cancer risk was observed either for radio users compared with non-users, or in relation to average monthly minutes of use (Supplementary Table 5).

The proportional hazards assumption was met in all the Cox models assessed.

## Discussion

In this study of over 48,000 police officers and staff across Great Britain, we found no evidence overall for an association between use of personal radio and risk of all cancers combined. A lower risk of all cancers and for cancers of the head and neck in users compared with non-users was accompanied by wide confidence intervals reflecting the limited follow-up time and numbers of cancers accrued into the cohort to date. In a sensitivity analysis restricted to forces with at least 5% linkage to CDR records, there was a weak association between radio use and lower all cancer risk among officers. The inverse association between radio use and cancer risk may reflect possible selection effects since non-users were older, heavier drinkers of alcohol, and more obese. At this stage, we are able to reliably exclude effects of TETRA use on all cancer risk (up to 1.25-fold) and risk of head and neck cancers (up to two-fold).

Evidence regarding the putative association between RF-EMF from mobile phones and cancer incidence has been extensively reviewed, including cellular, animal experimental, and epidemiological evidence. Overall, these studies have not shown consistent evidence of effect.^1,7–9^ The largest of the epidemiological studies, INTERPHONE, reported an excess of glioma and meningioma in the top decile of cumulative use of mobile phones,^7^ and excess risk of brain cancers was also reported in a series of case–control studies in Sweden.^10^ Recent results in animal models have strengthened the evidence for a putative carcinogenic effect of RF-EMF exposure^11,12^, but there is no clear biological mechanism for RF-EMF in cancer development,^13,14^ and the effects of RF-EMF on calcium efflux has also been disputed since the Stewart Report.^15,16^ Epidemiological studies of occupational RF-EMF exposure on risk of cancer have not shown any consistent or convincing evidence of effect,^1,17^ and recent in vitro work reported no effect of TETRA on electrophysiology of neuronal networks.^18^ Studies of acute effects of TETRA on the electroencephalogram were inconsistent^19–21^ as was an effect on heart rate variability, which was reported in one study (consistent with vagal stimulation in the chest)^19^ but not another which looked at head exposure.^22^ Other studies have concluded that there was no robust evidence for an effect of TETRA on cognitive functioning.^23,24^

The main strengths of our study are its size, prospective design, and availability of estimated TETRA use for cohort participants based on objective data, information on personal radio use from questionnaire, and participant characteristics that informed our imputation model.^6^ The principal limitation is the relatively small number of incident cancers to date, especially for head and neck cancers which are most relevant to TETRA use given their proximity to the handset, leading to imprecise estimates. Continued follow-up of the cohort over the next several years will allow further cases to accrue with concomitant improvement in precision. As another limitation, the influence of mobile phone use on cancer risks could not be adequately assessed as we only obtained a limited amount of self-report information without access to operator provided data to correct misreporting, similar to our TETRA approach.^6^ The use of objective TETRA usage data also had limitations, as for some radio users the average exposure before enrolment was calculated from only a few months of data, and we were unable to link CDR records for the largest force enrolled (Metropolitan police, ~8000 participants). Furthermore, we did not consider post-enrolment exposure as such analysis would be restricted to the subset with CDR records only, and there is potential for bias since ill-health may have affected post-enrolment usage patterns.

In summary, we found no evidence overall that TETRA use is associated with risk of cancer. Longer follow-up and, if data become available, pooling across studies are required to increase the number of cases and better estimate possible associations with cancer risk.

## Supplementary information

Supplementary material

## **Availability of data and material:**

Supplementary information is available at the British Journal of Cancer’s website
