# Supplementary material for: Personal radio use and cancer risks among 48,518 British police officers and staff from the Airwave Health Monitoring Study
Source: Br J Cancer. 2018 Dec 26;120(3):375–8. doi: 10.1038/s41416-018-0365-6 (PMC6354010; doi:10.1038/s41416-018-0365-6)

**SUPPLEMENTARY MATERIAL**

**Personal radio use and cancer risks among 48,518 British police officers and staff from the Airwave Health Monitoring Study**

He Gao, Maria Aresu**,** Anne-Claire Vergnaud, Dennis McRobie, Jeanette Spear, Andy Heard, Håvard Wahl Kongsgård, Deepa Singh**,** David C Muller**,** Paul Elliott

**Supplementary Tables**

**Supplementary Table 1**. Participant characteristics at study enrolment (N = 48,518)

**Supplementary Table 2:** Number of cancer cases by ICD-10 code groups among 48,518 participants from the Airwave Health Monitoring Study

**Supplementary Table 3:** Hazard ratios of personal radio use for all cancers after excluding forces with less than 5% of objective data on use among personal radio users

**Supplementary Table 4:** Characteristics of police officers by personal radio use status after excluding forces with less than 5% of objective data on use among personal radio users (N=24,403)

**Supplementary Table 5**: Hazard ratios of personal radio use for all cancers based on average radio usage before enrolment for users linked to their CDR records

**Supplementary Figure**

**Supplementary Figure 1**. Sample selection for the analyses

**Supplementary Tables**

**Supplementary Table 1. Participant characteristics at study enrolment (N = 48,518)**

|  | N (%) |  | N (%) |
| --- | --- | --- | --- |
| Sex |  | **Job satisfaction** |  |
| Female | 17,725 (36.5) | Dissatisfied | 6,669 (13.7) |
| Male | 30,793 (63.5) | Satisfied | 29,544 (60.9) |
| Personal radio user |  | Missing | 12,305 (25.4) |
| No | 16,149 (33.3) | **Body mass index*** |  |
| Yes | 32,369 (66.7) | Normal weight | 11,652 (24.0) |
| Rank |  | Overweight | 17,204 (35.5) |
| Officer | 31,255 (64.4) | Obese | 7,982 (16.5) |
| Staff | 11,766 (24.3) | Missing | 11,680 (24.1) |
| Missing | 5,497 (11.3) | **Alcohol drinking^#^** |  |
| Region |  | Past | 2,859 (5.9) |
| England | 33,943 (70.0) | Light and never | 20,133 (41.5) |
| Wales | 6,979 (14.4) | Moderate | 11,767 (24.3) |
| Scotland | 7,596 (15.7) | Heavy | 7,589 (15.6) |
| Education |  | Missing | 6,170 (12.7) |
| Vocational qualifications | 2,656 (5.5) | **Smoking** |  |
| GCSE equivalent or below | 12,563 (25.9) | Current | 5,089 (10.5) |
| A levels / Highers or equivalent | 11,908 (24.5) | Former | 10,588 (21.8) |
| Bachelor / Postgraduate degree | 10,362 (21.4) | Never | 31,972 (65.9) |
| Missing | 11,029 (22.7) | Missing | 869 (1.8) |
| Salary |  | **Daily number of cigarettes** |  |
| Less than £26,000 | 8,020 (16.5) | 0 | 31,972 (65.9) |
| £26,000-31,999 | 6,619 (13.6) | 1-4 | 1,376 (2.8) |
| £32,000-37,999 | 8,643 (17.8) | 5-9 | 3,090 (6.4) |
| More than £38,000 | 13,372 (27.6) | 10-15 | 4,250 (8.8) |
| Missing | 11,864 (24.5) | More than 15 | 6,371 (13.1) |
|  |  | Missing | 1,459 (3.0) |

GCSE, General Certificate of Secondary Education

Body mass index (BMI) was missing if a participant did not attend the health screening. Other missing values were due to no assessment of those participant characteristics in a specific version of the questionnaire or no response received from participants.

^*^ BMI was calculated as weight / height² and classified as: Normal weight, BMI<25kg/m^2^; overweight, 25<=BMI<30kg/m^2^; obese, BMI>=30kg/m^2^.^#^ Alcohol drinking was based on alcohol units calculated from different type of drinks/beverages consumed in the past week using sex-specific cut offs: light and never, <11 units in men or <7 in women; moderate, <=22 in men or <=15 in women; heavy, >22 in men or >15 in women.

**Supplementary Table 2: Number of cancer cases by ICD-10 code groups among 48,518 participants from the Airwave Health Monitoring Study**

| **ICD10 code** | **Description** | **N (%)** | **Head and neck** |
| --- | --- | --- | --- |
| C01 | MN of base of tongue | 5 (0.7) | √ |
| C02 | MN of other and unspecified parts of tongue | 3 (0.4) | √ |
| C06 | MN of other and unspecified parts of mouth | 1 (0.1) | √ |
| C07 | MN of parotid gland | 2 (0.3) | √ |
| C09 | MN of tonsil | 7 (1.0) | √ |
| C10 | MN of oropharynx | 3 (0.4) | √ |
| C11 | MN of nasopharynx | 1 (0.1) | √ |
| C13 | MN of hypopharynx | 1 (0.1) | √ |
| C15 | MN of oesophagus | 5 (0.7) |  |
| C16 | MN of stomach | 7 (1.0) |  |
| C17 | MN of small intestine | 1 (0.1) |  |
| C18 | MN of colon | 44 (6.2) |  |
| C19 | MN of rectosigmoid junction | 4 (0.6) |  |
| C20 | MN of rectum | 23 (3.2) |  |
| C21 | MN of anus and anal canal | 2 (0.3) |  |
| C22 | MN of liver and intrahepatic bile ducts | 1 (0.1) |  |
| C24 | MN of other and unspecified parts of biliary tract | 4 (0.6) |  |
| C25 | MN of pancreas | 6 (0.8) |  |
| C30 | MN of nasal cavity and middle ear | 1 (0.1) | √ |
| C32 | MN of larynx | 3 (0.4) | √ |
| C34 | MN of bronchus and lung | 16 (2.2) |  |
| C38 | MN of heart, mediastinum, and pleura | 1 (0.1) |  |
| C41 | MN of bone and articular cartilage of other and unspecified sites | 1 (0.1) |  |
| C43 | Malignant melanoma of skin | 85 (11.9) |  |
| C45 | Mesothelioma | 2 (0.3) |  |
| C48 | MN of retro peritoneum and peritoneum | 2 (0.3) |  |
| C49 | MN of other connective and soft tissue | 7 (1) |  |
| C50 | MN of breast | 139 (19.4) |  |
| C51 | MN of vulva | 3 (0.4) |  |
| C53 | MN of cervix uteri | 17 (2.4) |  |
| C54 | MN of corpus uteri | 18 (2.5) |  |
| C56 | MN of ovary | 11 (1.5) |  |
| C57 | MN of other and unspecified female genital organs | 1 (0.1) |  |
| C60 | MN of penis | 2 (0.3) |  |
| C61 | MN of prostate | 113 (15.8) |  |
| C62 | MN of testis | 19 (2.7) |  |
| C63 | MN of other and unspecified male genital organs | 2 (0.3) |  |
| C64 | MN of kidney, except renal pelvis | 27 (3.8) |  |
| C65 | MN of renal pelvis | 1 (0.1) |  |
| C67 | MN of bladder | 11 (1.5) |  |
| C69 | MN of eye and adnexa | 3 (0.4) | √ |
| C71 | MN of brain | 22 (3.1) | √ |
| C72 | MN of spinal cord, cranial nerves, and other | 1 (0.1) | √ |
| C73 | MN of thyroid gland | 21 (2.9) | √ |
| C80 | MN without specification of site | 1 (0.1) |  |
| C81 | Hodgkin's disease | 10 (1.4) |  |
| C82 | Follicular [nodular] non-Hodgkin's lymphoma | 9 (1.3) |  |
| C83 | Diffuse non-Hodgkin's lymphoma | 16 (2.2) |  |
| C84 | Peripheral and cutaneous T-cell lymphomas | 2 (0.3) |  |
| C85 | Other and unspecified types of non-Hodgkin's lymphoma | 7 (1.0) |  |
| C88 | Malignant immunoproliferative diseases | 1 (0.1) |  |
| C90 | Multiple myeloma and malignant plasma cell neoplasms | 6 (0.8) |  |
| C91 | Lymphoid leukaemia | 7 (1.0) |  |
| C92 | Myeloid leukaemia | 7 (1.0) |  |
| C93 | Monocytic leukaemia | 1 (0.1) |  |
| Total |  | 716 | 74 |

MN malignant neoplasm

**Supplementary Table 3: Hazard ratios of personal radio use for all cancers^#^ after excluding forces with less than 5% of objective data on use among personal radio users**

|  | All (N=38,888) | | |  | Males (N=24,181) | | |  | Females (N=14,707) | | |
| --- | --- | --- | --- | --- | --- | --- | --- | --- | --- | --- | --- |
|  | **N cases** | **HR (95% CI)** | **P-value** |  | **N cases** | **HR (95% CI)** | **P-value** |  | **N cases** | **HR (95% CI)** | **P-value** |
|  |  |  |  |  |  |  |  |  |  |  |  |
| Non-user | 315 | 1.00 |  |  | 139 | 1.00 |  |  | 176 | 1.00 |  |
| User | 334 | 1.04 (0.78-1.38) | 0.801 |  | 224 | 0.87 (0.60-1.25) | 0.443 |  | 110 | 1.24 (0.78-1.98) | 0.362 |
| Doubling of minutes of use |  | 0.98 (0.93-1.04) | 0.556 |  |  | 0.99 (0.92-1.06) | 0.702 |  |  | 1.01 (0.92-1.11) | 0.873 |
|  |  | | |  |  | | |  |  | | |
|  | **All officers (N=24,403)** | | |  | **Male officers (N=18,071)** | | |  | **Female officers (N=6,332)** | | |
|  |  |  |  |  |  |  |  |  |  |  |  |
| Non-user | 68 | 1.00 |  |  | 47 | 1.00 |  |  | 21 | 1.00 |  |
| User | 212 | 0.67 (0.45-1.00) | 0.049 |  | 151 | 0.64 (0.39-1.04) | 0.073 |  | 61 | 0.80 (0.39-1.63) | 0.534 |
| Doubling of minutes of use |  | 1.02 (0.96-1.09) | 0.518 |  |  | 1.02 (0.94-1.11) | 0.639 |  |  | 1.03 (0.91-1.16) | 0.631 |

HR, Hazard ratio

^#^ All cancers include all sites except for non-malignant melanoma (ICD10=C44).

The model adjusted for age (the underlying time-scale), sex, region, education, salary, rank, job satisfaction, BMI, smoking, number of cigarettes smoked, and alcohol drinking. The hazard ratio (HR) for usual personal radio use represents the increase in risk for a doubling of personal radio use (average number of minutes per month).

**Supplementary Table 4: Characteristics of police officers by personal radio use status after excluding forces with less than 5% of objective data on use among personal radio users (N=24,403)**

| **N (%)** | **All** | **User** | **Non-user** | **P-value** |
| --- | --- | --- | --- | --- |
|  | N=24,403 | N=21,348 | N=3,055 |  |
| **Age, mean (SD)** | 39.4 (7.9) | 38.7 (7.8) | 44.4 (7.1) | <0.001 |
| **Sex** |  |  |  | <0.001 |
| Female | 6,332 (25.9) | 5,322 (25.0) | 1,010 (33.1) |  |
| Male | 18,071 (74.1) | 16,026 (75.0) | 2,045 (67.0) |  |
| **Region** |  |  |  | <0.001 |
| England | 15,190 (62.2) | 13,064 (61.2) | 2,126 (69.6) |  |
| Scotland | 5,992 (24.6) | 5,390 (25.3) | 602 (19.7) |  |
| Wales | 3,221 (13.2) | 2,894 (13.5) | 327 (10.7) |  |
| **Education** |  |  |  | <0.001 |
| Vocational qualifications | 1,332 (5.5) | 1,199 (5.6) | 133 (4.4) |  |
| GCSE equivalent or below | 6,703 (27.5) | 5,741 (26.8) | 962 (31.5) |  |
| A levels / Highers or equivalent | 6,970 (28.6) | 6,155 (28.8) | 815 (26.7) |  |
| Bachelor / Postgraduate | 5,473 (22.4) | 4,804 (22.6) | 669 (21.9) |  |
| Missing | 3,925 (16.1) | 3,449 (16.2) | 476 (15.6) |  |
| **Salary** |  |  |  | <0.001 |
| less than £26,000 | 1,938 (7.9) | 1,729 (8.1) | 209 (6.8) |  |
| £26,000 - £31,999 | 4,341 (17.8) | 4,098 (19.3) | 243 (7.9) |  |
| £32,000 - £37,999 | 6,472 (26.5) | 5,687 (26.6) | 785 (25.7) |  |
| More than £38000 | 7,213 (29.6) | 5,937 (27.8) | 1,276 (41.8) |  |
| Missing | 4,439 (18.2) | 3,897 (18.3) | 542 (17.7) |  |
| **Body mass index*** |  |  |  | <0.001 |
| Normal weight | 5,639 (23.1) | 5,038 (23.7) | 601 (19.7) |  |
| Overweight | 10,061 (41.2) | 8,823 (41.4) | 1,238 (40.5) |  |
| Obese | 4,353 (17.8) | 3,670 (17.2) | 683 (22.3) |  |
| Missing | 4,350 (17.8) | 3,817 (17.9) | 533 (17.5) |  |
| **Alcohol drinking^#^** |  |  |  | <0.001 |
| Past | 1,368 (5.6) | 1,191 (5.6) | 177 (5.8) |  |
| Light and never | 10,594 (43.4) | 9,494 (44.4) | 1,100 (36.0) |  |
| Moderate | 6,655 (27.3) | 5,807 (27.2) | 848 (27.7) |  |
| Heavy | 4,436 (18.2) | 3,698 (17.3) | 738 (24.2) |  |
| Missing | 1,350 (5.5) | 1,158 (5.4) | 192 (6.3) |  |
| **Smoking** |  |  |  | <0.001 |
| Never | 16,785 (68.8) | 14,803 (69.3) | 1,982 (64.9) |  |
| Former | 5,038 (20.6) | 4,294 (20.1) | 744 (24.3) |  |
| Current | 2,322 (9.5) | 2,013 (9.5) | 309 (10.1) |  |
| Missing | 258 (1.1) | 238 (1.1) | 20 (0.7) |  |
| **Daily number of cigarettes** |  |  |  | <0.001 |
| 0 | 16,785 (68.8) | 14,803 (69.3) | 1,982 (64.9) |  |
| 1-4 | 581 (2.4) | 508 (2.4) | 73 (2.4) |  |
| 5-9 | 1,468 (6.0) | 1,317 (6.2) | 151 (5.0) |  |
| 10-15 | 2,026 (8.3) | 1,736 (8.1) | 290 (9.5) |  |
| More than 15 | 3,005 (12.3) | 2,503 (11.8) | 502 (16.4) |  |
| Missing | 538 (2.2) | 481 (2.2) | 57 (1.9) |  |
| **Job satisfaction** |  |  |  | <0.001 |
| Dissatisfied | 3,274 (13.4) | 2,948 (13.9) | 326 (10.7) |  |
| Satisfied | 16,365 (67.1) | 14,203 (66.6) | 2,162 (70.7) |  |
| Missing | 4,764 (19.5) | 4,197 (19.7) | 567 (18.6) |  |

GCSE, General Certificate of Secondary Education

Body mass index (BMI) was missing if a participant did not attend the health screening. Other missing values were due to no assessment of those participant characteristics in a specific version of the questionnaire or no response received from participants.

^*^ BMI was calculated as weight / height² and classified as: Normal weight, BMI<25kg/m^2^; overweight, 25<=BMI<30kg/m^2^; obese, BMI>=30kg/m^2^.

^#^ Alcohol drinking was based on alcohol units calculated from different type of drinks/beverages consumed in the past week using sex-specific cut offs: light and never, <11 units in men or <7 in women; moderate, <=22 in men or <=15 in women; heavy, >22 in men or >15 in women.

**Supplementary Table 5: Hazard ratios of personal radio use for all cancers^#^ based on average^&^ radio usage before enrolment for users linked to their CDR records**

|  | All (N=32,330) | | |  | Males (N=19,572) | | |  | Females (N=12,758) | | |
| --- | --- | --- | --- | --- | --- | --- | --- | --- | --- | --- | --- |
|  | **N cases** | **HR (95% CI)** | **P-value** |  | **N cases** | **HR (95% CI)** | **P-value** |  | **N cases** | **HR (95% CI)** | **P-value** |
|  |  |  |  |  |  |  |  |  |  |  |  |
| Non-user | 315 | 1.00 |  |  | 139 | 1.00 |  |  | 176 | 1.00 |  |
| User | 259 | 1.17 (0.82-1.65) | 0.302 |  | 176 | 1.08 (0.70-1.67) | 0.717 |  | 83 | 1.15 (0.63-2.14) | 0.713 |
| Doubling of average minutes of use |  | 0.97 (0.91-1.03) | 0.390 |  |  | 0.96 (0.89-1.04) | 0.311 |  |  | 1.02 (0.91-1.15) | 0.645 |
|  |  | | |  |  | | |  |  | | |
|  | **All officers (N=19,647)** | | |  | **Male officers (N=14,566)** | | |  | **Female officers (N=5,081)** | | |
|  |  |  |  |  |  |  |  |  |  |  |  |
| Non-user | 68 | 1.00 |  |  | 47 | 1.00 |  |  | 21 | 1.00 |  |
| User | 177 | 0.72 (0.45-1.16) | 0.181 |  | 125 | 0.75 (0.42-1.34) | 0.336 |  | 52 | 0.71 (0.29-1.74) | 0.454 |
| Doubling of average minutes of use |  | 1.01 (0.93-1.10) | 0.810 |  |  | 0.99 (0.90-1.10) | 0.882 |  |  | 1.06 (0.90-1.23) | 0.501 |

HR, Hazard ratio; CDR, Caller data record

^#^ All cancers include all sites except for non-malignant melanoma (ICD10=C44).

^&^ During last consecutive period of TETRA usage before enrolment if there is a gap in use.

The model adjusted for age (the underlying time-scale), sex, region, education, salary, rank, job satisfaction, BMI, smoking, number of cigarettes smoked, and alcohol drinking. The hazard ratio (HR) for usual personal radio use represents the increase in risk for a doubling of personal radio use (average number of minutes per month).

**Supplementary Figure 1.** **Sample selection for the analyses**


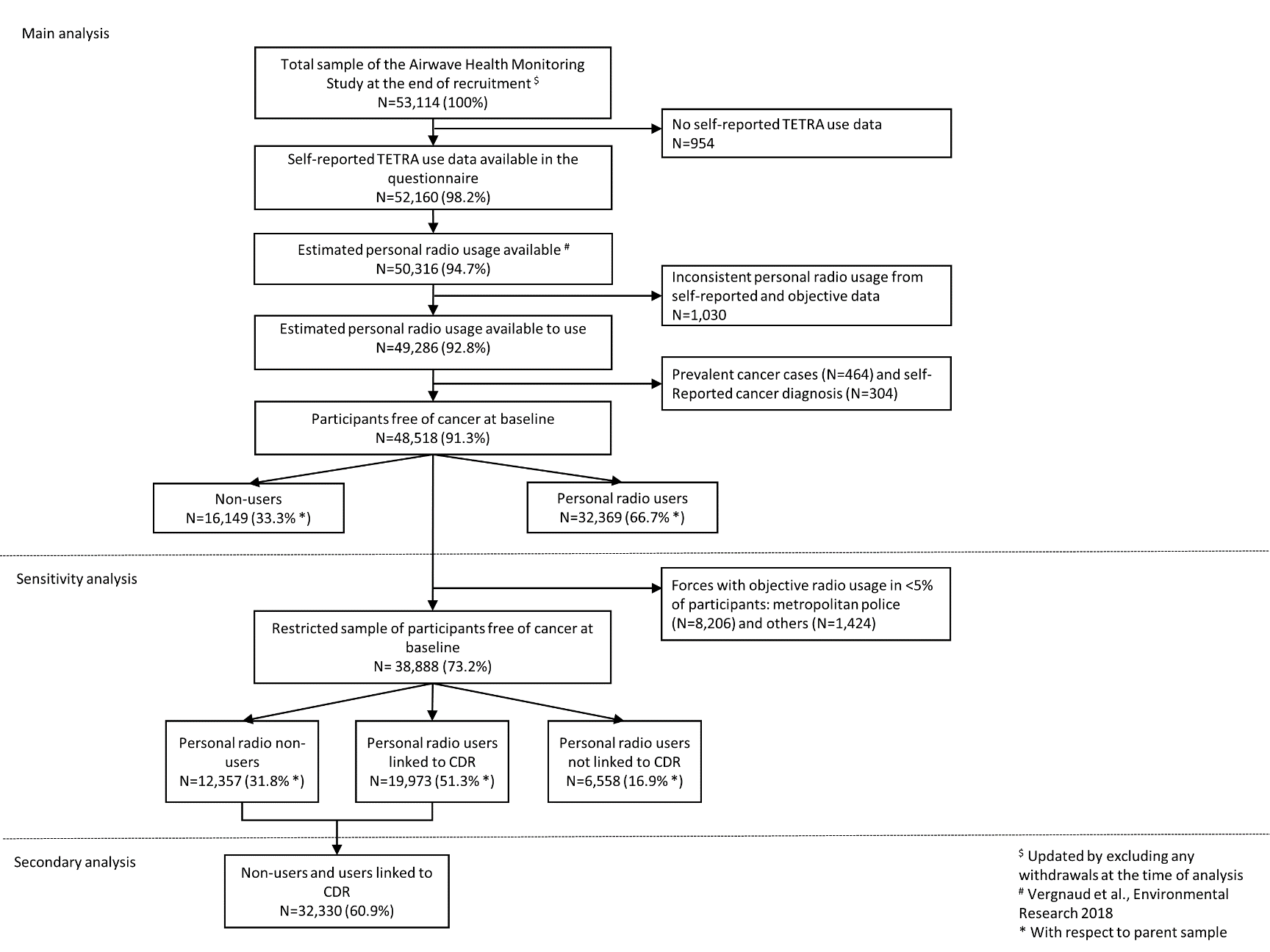

Supplement: Supplementary file 1 — Supplementary material [file 41416_2018_365_MOESM1_ESM.docx]
